# Necrotising Enterocolitis in Preterm Infants: Epidemiology and Antibiotic Consumption in the Polish Neonatology Network Neonatal Intensive Care Units in 2009

**DOI:** 10.1371/journal.pone.0092865

**Published:** 2014-03-21

**Authors:** Jadwiga Wójkowska-Mach, Anna Różańska, Maria Borszewska-Kornacka, Joanna Domańska, Janusz Gadzinowski, Ewa Gulczyńska, Ewa Helwich, Agnieszka Kordek, Dorota Pawlik, Jerzy Szczapa, Piotr B. Heczko

**Affiliations:** 1 Chair of Microbiology, Jagiellonian University Medical College, Kraków, Poland; 2 Clinic of Neonatology and Intensive Neonatal Care, Warsaw Medical University, Warsaw, Poland; 3 Institute of Theoretical and Applied Informatics of the Polish Academy of Sciences, Gliwice, Poland; 4 Chair and Department of Neonatology, Poznań University of Medical Sciences, Poznań, Poland; 5 Clinic of Neonatology, Polish Mother's Memorial Hospital-Research Institute, Łódź, Poland; 6 Clinic of Neonatology and Intensive Neonatal Care, Institute of Mother and Child, Warsaw, Poland; 7 Department of Neonatal Diseases, Pomeranian Medical University, Szczecin, Poland; 8 Clinic of Neonatology, Jagiellonian University Medical College, Kraków, Poland; The Ohio State Unversity, United States of America

## Abstract

The aim of this study was to describe the epidemiology of necrotising enterocolitis (NEC), antibiotic consumption and the usefulness of microbiological tests in very low birth weight (VLBW) Polish newborns.

**Methods:**

Prospective surveillance was performed in the year 2009 by local infection control teams. The study covered 910 infants hospitalized in six Polish neonatal intensive care units. Two kinds of indicators were used for the description of antibiotic usage: the duration of treatment (days of treatment, DOTs) and the defined daily dose (DDD).

**Results:**

NEC incidence was 8.7% and fatality rate was 19%. Chorioamnionitis, late gestational age and low birth weight were identified as risk factors for NEC. Catheterization, mechanical ventilation and other selected procedures were used considerably longer in newborns with NEC than in the remaining neonates. Total usage of antibiotics reached 2.9 DDDs or 1.437 days; the average use of drugs per case of NEC amounted to 0.47 DDD or 23.2 DOTs. The level of antibiotic usage was analysed with correlation to microbiological tests performed and it was non-significantly greater in the group of children with NEC in whom the tests were performed.

**Conclusions:**

A high risk of developing NEC is closely associated with VLBW and with inflammation of the amnion during labour. We observed no relationship between the consumption of antibiotics in neonates with NEC and positive results of microbiological testing indicating sepsis accompanying NEC or gut colonization with pathogens.

## Introduction

Necrotising enterocolitis (NEC) is one of the most unpredictable and devastating diseases in premature infants. To date there is no one theory as to the etiology of NEC; however, most researchers agree that the pathogenesis is multifactorial and has often been associated with enteral feedings, bowel ischemia and infectious causes [1]. The process leading to NEC is thought to be preceded by an ischemic or toxic event that causes damage to the immature gastrointestinal mucosa and loss of mucosal integrity [2]. The initiation of enteral feedings allows for bacterial proliferation at which time the damaged mucosa is invaded by gas-producing bacteria. This process may lead to necrosis which can cause either perforation of the bowel or sepsis [3] and then may often lead to sepsis caused by translocation of gut colonizing nosocomial pathogens in the peritoneum and/or blood [4].

With an incidence of 6–7%, NEC is also one of the leading causes of mortality among infants [5]–[8]. It is a big and costly problem especially for infants who are born with very low birth weight (VLBW). In a study by Ganapathy and co-workers, the adjusted incremental costs of NEC were more than $198,000 (U.S. dollars) per infant in their 2012 report [9].

The aim of the study was to investigate the incidence rate of NEC among VLBW neonates, determine selected risk factors accompanying sepsis and gut colonization by nosocomial pathogens, and assess the level of antibiotic usage and its correlation to performing microbiological tests.

## Methods

Electronic database created as the result of continuous prospective targeted surveillance of infections was used in the study. Data were collected between 1.01.2009 and 31.12.2009 in six tertiary academic neonatal intensive care units (NICU) that took part in the Polish Neonatology Surveillance Network (PNSN). The PNSN is a prospective national surveillance system for the most relevant infections in VLBW infants (birthweight < 1500 g) in Poland. Details of the following variables were collected for all VLBW newborns: birthweight and gestational age, gender, multiple pregnacy, type of delivery and information of the situation in time of delivery, for example chorioamnionitis, general status of newborns by Apgar score: at 1 and 5 minutes and Critical Risk Index for Babies, CRIB and others). The PNSN recorded severe infections, including necrotizing enterocolitis observed in the time on hospitalization: from admission to discharge, transfer or death. Participation in PNSN was voluntary and confidential. Utilization of data collected in PNSN for the scientific purpose was approved by the Bioethics Committee of Jagiellonian University Medical College – no. KBET/221/B/2011. All data entered into the electronic database and analyzed during preparing this article were previously anonymized and de-identified. Those data were obtained under routine treatment and diagnostic procedures performed during patients' hospitalization. According to Polish law, utilization this kind of data for scientific purpose does not demand patients' agreement or even information that data are collected in the database. Before analysis incomplete records were deleted and as the result all records of 485 newborns without NEC and 79 NEC cases were filled with all data.

Thus, the utilization of those data for the analysis is consistent with the Polish law and approved by the institutional University bioethics committee.

Infants with birth weight lower than 1,500 grams were included in the study. Case NEC patients were defined according to Gastmeier et al [10] as neonates with VLBW who had clinical symptoms of necrotising enterocolitis (NEC), i.e., at least two of the following signs: vomiting, abdominal distention, pre-feeding residuals, redness of flanks, persistent microscopic or gross blood in stools; and at least one of the following criteria: pneumoperitoneum, pneumatosis intestinalis, unchanging ‘rigid’ loops of small bowel; or histological evidence of NEC, considered as infection.

Standard indicators of general status of newborns was Apgar score (at 1 and 5 minutes) and Critical Risk Index for Babies (CRIB). Chorioamnionitis diagnosis was based on clinical evaluation without the need for microbiological or histopathological examination [11].

The amount of human milk provided in natural feeding was not described in study protocol in a quantitative manner.

Microbiological tests were performed in some neonates according to doctor's orders and the presence of clinical symptoms of NEC. Blood or rectal swabs were collected (any signs of NEC) for culture and assessment of the presence of facultative pathogens. The specimens were cultured on McConkey agar, horse blood agar and Sabouraud agar (37°C, 24 h each). Identification with API tests (bioMerieux) was then performed.

Antibiotic usage for NEC treatment (until cure) was assessed for 59 cases; eight cases were excluded from analysis due to death of the infants in the first seven days of NEC (unsuccessful treatment). The elimination of one case was connected with an internal validation of data (extremely high consumption of antibiotics, i.e., more than 10 times greater than in other cases, suggesting recording bias). In other cases (nine newborns), data about treatment were not available. Two kinds of indicators were used for the description of antibiotic usage: the duration of treatment (days of treatment, DOTs) expressed in days and the defined daily dose (DDD), according to the ATC/DDD system of the World Health Organization (Anatomical Therapeutic Chemical, group “J01”), both measures in reference to one case of infection.

For the evaluation of the differences between the averages for the examined groups of infants (the examined group with NEC vs the infants without any infections), one-way analysis of variances (ANOVA) with the least significant difference (LSD) test and the Tukey's test were applied. For the assessment of the frequency of infections in various groups of infants, the chi-square test of independence was used. A nonparametric Mann-Whitney U test was applied to assess the significance of differences between the length of antimicrobial treatment of infections or the number of DDD and positive result of microbiological tests. The calculations were performed with the application of the SciPy open source library; the assumed significance level was p<0.05.

## Results

### Characteristics of study population

The activities of the PNSN covered 910 VLBW newborns including 166 extremely low birth weight infants (18.2%) with a birth weight up to 750 grams, 250 infants (27.5%) with birth weight ranging from 751 to 999 grams and 494 infants (54.3%) with birth weight ranging from 1,000 to 1,500 grams. The number of VLBW newborns covered by the survey accounted for 19.1% of all VLBW infants born in Poland in 2009.

Their mortality rate within the entire country of Poland was 24.3%, while in the examined units it averaged 19%^12^ Overall, 812 deliveries were described, 223 infants were born from multiple pregnancies. All wards had perinatal care and were in the tertiary referral teaching hospitals.

The most frequently observed symptoms of NEC were: abdominal distension (98.5% of cases), pre-feeding residuals/gastric retention (97.5%), impaired intestinal motility (79.4%), elevated C-reactive protein (73.5%) and radiographic signs (33.8%).

Among the hospitalised infants, 425 had at least one infection; 79 infants had symptoms of NEC. The NEC incidence was 8.7%, the risk of NEC development did not differ between centres. Thus, newborns with NEC accounted for 12.7% of all, that was 625 cases of infection.

### Risk Factor Analysis

Among the children with NEC, 63 (79.7%) were breastfed. One NEC case was observed among infants who were not provided with total parenteral nutrition (TPN), incidence rate – 1.7%. The NEC symptoms in this case were observed at the 27^th^ day of life of the neonate born with a birth weight of 1,480 grams.

The mean gestational age and birth weight of the premature with NEC were significantly lower (median: 27) than the mean gestational age in the group of newborns without any infections (median: 28) (Table 1); the significantly higher incidence rate (11.8%) was confirmed in the group of infants with birth weight less than 1,000 grams (relative risk, RR 1.9).

**Table 1:** Characteristics of neonates with symptoms of NEC and newborns without signs of any infections.

	Number of newborns				
	Without NEC or others infetciotns [N = 485]		With NEC [N = 79]		
	mean	95%CI	mean	95%CI	p- value
Gestational age [week]	28	28; 29	27	26; 27	<0.001
Birth weight [gram]	1 058	1 029; 1 087	918	861; 974	<0.001
**Participated in the study group**	**N**	**%**	**N**	**%**	
Female	233	48.0	32	40.5	–
Caesarean sections	339	69.9	55	69.6	–
Single pregnancy	340	70.1	70	88.6	<0.001
Trophic feeding	181	37.3	35	44.3	–
**CRIB** *	**N**	**%**	**N**	**%**	
0–4	101	20.8	12	15.2	–
5–10	26	5.4	7	8.9	–
11–15	10	2.1	2	2.5	–
15–22	8	1.6	0	0.0	–
**Apgar (1 min)**	**N**	**%**	**N**	**%**	
0–4	183	37.7	29	36.7	–
5–7	199	41.0	35	44.3	–
8–10	103	21.1	15	19.0	–
**Apgar (5 min)**	**N**	**%**	**N**	**%**	
0–4	129	26.6	22	27.8	–
5–7	201	41.4	39	49.4	–
8–10	155	32.0	18	22.8	–
PROM	106	21.9	22	27.8	–
Chorioamnionitis diagnosed during pregnancy	26	5.4	9	11.4	–
Fatal cases	120	24.7	16	20.3	–

CI – confidence interval; CRIB – Clinical Risk Index for Babies; NEC – necrotizing enterocolitis; PROM – prolonged rupture of membranes;

*CRIB index was calculated and used °nly in two of six NICUs taking part in this analysis.

Statistical analysis of the relationship between the occurrence of NEC and chorioamnionitis diagnosed in mothers showed that this relationship was statistically significant (p<0.005), but the RR and 95% CI values did not allow an unambiguous assessment of this relationship (RR 2.1, 95% confidence interval, CI 0.9143-4.4092).

The gender of the infants did not influence the risk of NEC; neither did the general condition of the infant as expressed by the CRIB score and the APGAR score at 1 and 5 minutes after birth. The occurrence of a single vs multiple pregnancy (twin birth, triplet birth or more) decreased the risk of NEC (RR 0.01, 95% CI 1.4187-5.8781). The type of feeding, natural, trophic, or parenteral, did not influence the risk of NEC in studied group of VLBW infants (Table 1).

### Correlation between selected risk factors and observed outcomes

Newborns with NEC symptoms were more frequently vulnerable to late-onset pneumonia (according to Gastmeier et al [12]) (RR 2.2, 95% CI 1.930205.1491). Catheterisation, mechanical ventilation, antibiotic use and other selected procedures (as a consequence of NEC) were used for a significantly longer period of time in newborns with NEC (p<0.001) (Tab. 1) compared to neonates without any infections.

NEC was the direct cause of death in 18 newborns, fatality case rate was 22.8%.

### Microbiological assays in neonates with NEC and antibiotic consumption

Microbiological tests performed using the culture method for the determination of facultative pathogens were done in 85.2% of the cases. Presence of bacterial strains regarded as facultative pathogens in blood or rectal swabs was confirmed in 53% of samples. Gram-positive cocci were most frequently isolated and constituted 62.9% of all microorganisms; coagulase-negative staphylococci were the most often detected among them (42.9%). Gram-negative rods were isolated with a frequency of 34.3% (total); *Escherichia coli* – 11.4%, *Klebsiella spp* – 17.1% and *Enterobacter spp.* of 5.7% of all cases (Table 2). Yeasts constituted 2.9% of all cases.

**Table 2:** Microorganisms isolated from blood or rectal swabs of neonates with NEC.

	Type of material tested			
Species	Blood	Rectal swabs	Total	%
*Staphylococcus aureus*	2	0	3	5.7
Coagulase negative staphylococci	9	6	15	42.9
*Enterococcus spp.*	1	4	5	14.3
*Escherichia coli*	3	1	4	11.4
*Klebsiella spp.*	0	6	6	17.1
*Enterobacter spp.*	0	2	2	5.7
*Candida spp.*	0	1	1	2.9
**TOTAL**	**15**	**20**	**35**	**100.0**

In the analysed group, 29.4 DDDs of antibiotics were used for treatment of NEC and total treatment duration was 1,437 days (Table 3). Average usage of antimicrobials in all wards was 23.2 DOTs or 0.5 DDDs per case. The lowest antibiotic usage was observed in ward B – 9.27 DOTs or 0.2 DDDs; the highest in ward C – 46.0 DOTs or 0.9 DDDs (about four times higher). The differences observed were related to the monitoring of the infections carried out in NICUs, which was reflected in the fact that the overall incidence rate was twofold higher in ward C. The antibiotics most often used for treatment were beta-lactams, aminoglycosides, glycopeptides and metronidazole; the percentage of treatment duration for these groups of antimicrobials was 35.8%, 5%, 14.3% and 12.5%, respectively. The only antifungal drug used in the treatment was fluconazole, for 19% of the treatment days.

**Table 3:** Antibiotic usage in NEC cases according to studied NICUs.

Ward	DOTs [day]	DOTs per number of NEC	DDDs [no]	DDDs per number of NEC
A	98	12.25	0.3	0.3
B	102	9.27	0.2	0.2
C	322	46.00	0.6	0.9
D	218	27.25	0.4	0.5
E	376	31.33	1.0	0.8
F	321	20.06	4.3	0.3
TOTAL	1 437	23.18	29.4	0.5

NEC – necrotizing enterocolitis; DDD – defined daily dose; DOTs – days of treatment;

According to both indicators, i.e. duration of treatment and DDDs, antibiotic usage was non-significantly greater in the group of 38 infants with NEC in whom microbiological tests were not performed. In this group, the average usage of antibiotic treatment was 28.0 DOTs or 0.6 DDD per case. Antibiotic consumption was lower in the group of 21 NEC infants in whom microbiological tests were performed, and amounted to 18.6 DOTs or 0.4 DDD per one case of infection. Data concerning antimicrobial consumption in reference to microbiological tests in individual NICUs are presented in Table 4.

**Table 4:** Antibiotic usage in NEC cases with and without microbiology tests.

	DOTs [days]		DOTs per number of NEC		DDDs [no]		DDDs per number of NEC	
Ward	with*	without^**^	with*	without^**^	with*	without^**^	with*	without^**^
A	32	66	10.7	13.2	0.4	2.3	0.1	0.4
B	102	0	9.3	ns	0.2	0	0.2	ns
C	154	168	51.3	42.0	2.9	3.4	0.7	0.8
D	23	195	11.5	32.5	0.5	3.9	0.2	0.7
E	61	315	15.3	39.4	0.2	8.1	0.4	1.0
F	0	321	ns	20.1	0	4.3	ns	0.3
**TOTA**L	**372**	**1 065**	**18.6**	**28.0**	**7.5**	**21.9**	**0.4**	**0.6**

*cases of NEC with isolated microbiological agent; **cases of NEC without isolated microbiological agent;

DOTs – days of treatment; DDD – defined daily dose; NEC – necrotizing enterocolitis.

## Discussion

Our results of late-onset infections are the first to be reported by the PNSN and from Central (except NeoKISS) Europe based on a national programme for infection control in neonatal units. In Poland, fewer than 5000 VLBW infants are born each year, i.e., 0.9% of all live births [13].

In the literature, incidence attributed to NEC is assessed at a level of 6–7%, and the authors indicate a large variability of the reported values depending on the population studied [12], [14], [15]. According to Gagliardi, the highest incidence reaching 13%, was observed among newborns delivered in the 24^th^ week of pregnancy, while the incidence rate of NEC among infants delivered in the 27^th^ week or later was as low as 3% [16]. Thus, the mean gestational age of premature newborns in our population was 28 weeks (median: 27) suggesting that the incidence rate in the Polish population studies is higher than those observed in other studies.

Among the most important risk factors for developing NEC, the following are considered important: low Apgar score [17], the need for surfactant application, patent ductus arteriosus and late-onset bloodstream infections [16]. In the recent review on NEC [18], the following risk factors were also listed: prematurity (<28 weeks), enteral feeding (90% are fed enterally), growth restriction, maternal hypertensive disease of pregnancy, placental abruption, absent or reversed end diastolic flow velocity, use of umbilical catheters and packed cell transfusions. Such a great range of factors may indicate a lack of certainty referred to etiology of this clinical entity, although a proposed concept of NEC pathologic mechanisms [18] seems to indicate a progress in understanding its pathomechanisms.

The presented results indicate a close correlation between NEC incidence and chorioamnionitis. Thus, our findings are in concordance with a recent review by Been et al [19]. Their meta-analysis indicates an important role of antenatal inflammation in NEC development. This factor has not yet been analysed in detail, but may suggest a potential relationship between NEC and improper gut colonisation during the neonatal period, or at the moment of birth, which has already been indicated by other authors [20]–[22].

Other procedures, which have not been analysed in the present study but may influence NEC incidence, such as the use of probiotics [23], [24], L-arginine [25], lactoferrin [26] or the extended empirical antibiotic treatment [17] still require intensive multicentre studies.

Also, current trial data do not provide evidence that delayed introduction of progressive enteral feeds reduces the risk of NEC in VLBW infants [27].

Sepsis is an infection accompanying NEC. In the study published by Salbah et al [4], the proportion of infants with culture-proven nosocomial sepsis was higher in the NEC group than in controls without NEC. Of the organisms isolated from the NEC infants 50% were Gram-negative bacilli as compared to 14% in the controls; 44% of the organisms isolated from the NEC cases were Gram-positive cocci as compared to 72% in the controls. Fungal infections occurred in 6% of the NEC cases as compared to 14% in the controls [22]. We obtained very similar data from our observations, although in general Gram-positive cocci predominated over Gram-negative rods in cultures obtained from blood of the NEC group. Cultures from rectal swabs, which represent gut colonization, showed a predominance of Gram-negative rods over Gram-positive cocci. Coagulase negative staphylococci (CoNS) were the only Gram-positive microorganism isolated from rectal swabs and its role as a potential etiological factor of NEC was examined previously by others. Hoy et al noted the presence of CoNS in duodenal aspirates of VLBW infants, they are commonly found in the stools of NEC infants and have been associated with serious diseases [28]. The role of staphylococcal delta(δ)-toxin was examined by Scheifele et al and Scheifele and Bjornson, who believed that toxin positive CoNS were enteropathic. δ-toxin, a secreted protein with a detergent-like action, caused significant bowel necrosis in infant rats and was cytotoxic for fibroblasts in vitro [29], [30]. Moreover, it could be detected in the stools of infants colonised with δ-toxin producing CoNS. Rejection of the δ-toxin theory does not preclude a role for CoNS *per se*, which are known to express a number of other virulence factors. In addition, preterm infants exhibit deficiencies in immune responses to CoNS, suggesting they may cause more aggressive infections in this group compared to term neonates [31].

The problem of the diagnostic value of microbiological test results obtained during NEC is not often discussed at present by researchers. The reason for this may be diagnostic difficulties concerning the choice of clinical materials (samples) and microbiological tests that enable obtaining the results indicative for antibiotic treatment. At the moment, no recommendations in this area exist, since a possible microbial aetiology of NEC remains obscure. A good diagnostic material could definitely be, e.g., peritoneal fluid [32], but it is available only in patients in whom surgical intervention is required.

It is of interest that although various antibiotic regimens are commonly and widely used in the treatment of NEC, there is no sufficient evidence to recommend a particular antibiotic for its treatment [33]. Even though microbial aetiology remains unknown, preventing and treating sepsis with antibiotics yet avoiding prolonged exposure to them is equally important [34]. The results of this study confirm that bias: the utilisation of the microbiological diagnostic tests in neonates with NEC did not influence the level of antibiotic usage expressed by either DOTs or DDDs, the influence of results of the microbiological diagnostics on the scheme of treatment is unknown.

Creating and managing antibiotic policy is one of the main elements of the infection surveillance. The most commonly used measure of drug consumption is the defined daily dose, which is the assumed average maintenance dose per day of drug use for its main indication in adults. In premature, the direct measurement of the number of days of treatment (DOTs) may have a better application. Both measures were used in this study. Antibiotic usage in the treatment of NEC cases in hospitals taking part in the study varied both using the DOT or DDD rate per case, especially comparing the level of antibiotic usage in ward B and in ward C – where it was about four times higher. The differences observed were related to the monitoring of the infections carried out in NICUs, which was reflected in the fact that the overall incidence rate was twofold higher in ward C. This confirms that a lack of infection observation is also a lack of antibiotic consumption observation.

Average values of antibiotic usage in the group of NEC cases with microbiological support were non-significantly lower and reached 18.6 DOTs or 0.4 DDDs per case, contrary to the group of NEC cases without microbiological testing, where the usage was 28.0 or 0.6, respectively (about 70% higher). Due to the relatively small sample size, further research indicating the possibility of the application of microbiological diagnostics in neonates with NEC is necessary because of frequent sepsis cases caused by common hospital pathogens such as staphylococci and Gram-negative rods. Perhaps outcomes from a larger population would make it possible to obtain a result that justifies the inclusion of this element in the treatment of NEC. In cases of NEC, applications of microbiological diagnostics are very limited and there is a lack of studies on the effectiveness of the diagnostic tests on antibiotic usage. In the tested NECs, reliable microbiological tests were performed in a little over half of the cases.

In most of the NICUs, a shorter duration of antibiotic treatment was accompanied by lower antibiotic usage as expressed by the DDD rate, both when analyses were conducted for all cases of infection and in the case of dividing NEC patients into two groups, those with and without microbiological tests. The results of this data analysis of antibiotic consumption allow us to draw two conclusions: one regarding the choice of the method for benchmarking data between different hospitals, and the effectiveness of antibiotic treatment based on the results of microbiological tests in NEC cases.

Despite the limitation of DDD in regard to child populations, this kind of measure may be used for benchmarking purposes as well as the days of treatment (DOTs) rate; the level of antibiotic consumption and its differentiation in the two analysed groups in this study were similarly reflected by both indicators.

The shorter duration of antibiotic treatments and lower values of DDDs in each analysed ward in groups of NEC cases as compared to the group without microbiological examination confirmed the necessity and reasonability of routine microbiological testing to detect infections. Microbiological diagnostics in Polish hospitals are still regarded as expensive procedures and, consequently, are often performed only in cases of empiric treatment failure. However, a shorter treatment results in lower expenditures on antimicrobials, which should be taken into account during an analysis of the cost-effectiveness of microbiological testing for hospitalised patients.

However, irrespective of the test method, the antibiotic associated with NEC employed in the studied NICUs differs from the schemes suggested in literature [22].

The presented observation has also some limitations. The group studied, although diverse in terms of prevalence of risk factors for infection, includes only VLBW infants. The results would probably be more telling if the survey covered the entire population of NICU newborns. But the main limitation of our study is very low number of NEC cases, but this is due to the specific nature of the disease and the specific population, i.e., VLBW infants. Confirmation of significant methodological limitations is that the mortality associated with NEC in this study was not significantly lower than the mortality in the control group, mainly due to the high overall perinatal mortality of VLBW infants.

On the other hand, is NEC truly an infection? A clear interpretation is lacking.

## Conclusions

A high risk of developing NEC is closely associated with VLBW and with inflammation of the amnion during labour. We observed no relationship between the consumption of antibiotics in neonates with NEC and positive results of microbiological testing indicating sepsis accompanying NEC or gut colonization with pathogens. Whilst it is important to raise awareness of use of antibiotics without clear microbiological reason, for NEC this is common practice and it is difficult to imagine how this could be changed. Unfortunately, in this case there is no simple solution. Thus, further detailed studies on the purpose, and usefulness, of the microbiological diagnosis of sepsis and gut colonization by pathogens in order to rationalize the use of antibiotics in NEC seem to be desirable.
